# A triple pandemic: COVID-19, violence against children, and the crisis in family courts

**DOI:** 10.3389/frcha.2025.1481455

**Published:** 2025-03-31

**Authors:** Bandy X. Lee, Grace Lee

**Affiliations:** ^1^ Harvard Medical School, Boston, MA, United States; ^2^ Yale University, New Haven, CT, United States

**Keywords:** pandemic, COVID-19, violence against children, family courts, judicial reform

## Introduction

The COVID-19 pandemic has widely impacted the health and survival of populations worldwide. Despite severe disease and death remaining rare in children (1), children have been particularly vulnerable to upsurges in violence in the home (2, 3). Restrictions to limit the viral spread exacerbated the conditions for “twin pandemics.” Social factors further contributed through stress, poverty, unemployment, school closures, and lack of contact with support providers (4). Globally, violence against children was already at epidemic levels before COVID-19, with an estimated 1 billion children and adolescents experiencing abuse of some kind each year (5). Natural disasters and external stressors increase this risk (6, 7), and these incidents are furthermore closely linked to family violence and violence against women, which affects approximately one-third of women around the world (8).

However, yet another hidden but deadly scourge—the crisis in family courts—is as much an exacerbator as a side effect and requires urgent attention. Victims of violence may seek remedies through social services, including the family court system, believing that these can help reduce the harm (9, 10). In many countries, divorce, separation, and child custody issues are dealt with in family courts, with practices that deviate considerably from those of civil or criminal courts. This deviation, originally intended for the protection of children, has in the absence of transparency and accountability led to deadly results for minors.

## The statistics

A largely unknown, tragic fact is that family courts have become a place where too many vulnerable children die. A non-profit organization has tracked 990 child murders by a separating parent since 2008 in the United States (11). At the height of the COVID-19 pandemic, between the years 2020 and 2022, child murders by separating parents increased by 47.8% (11). An astonishing fact is that, instead of protecting these children, family court judges’ decisions facilitated many of these murders (11). A closer look at 175 child murders by fathers showed that, in many cases, the judges had ordered the conditions that allowed for the murders, often over the mothers' objections (12). An even greater number of suicides occur for every murder (13), and hundreds of injuries are medically treated for every death (14). Furthermore, the actual numbers are likely to be much higher, as the routine sealing of court records against the convention of open courts, in the name of the “protection of children,” has made tracking actual numbers of child murders as a result of family court decisions virtually impossible.

## The mechanism

How does this happen? In the US, family court judges have almost unlimited discretion, including the ability to seal court records, without oversight or accountability. A net result is that those with few rights, notably children, are violated in ways that multiply their harm. The vast majority of the approximately 100,000 contested child custody cases per year are family violence cases. Abusive fathers disproportionately seek sole custody in these cases, unlike trends outside of family court, and family courts grant it almost 75% of the time (15). A report in the United Kingdom, the first country to compel the opening of family courts to reporters, found that even convicted child rapists were sometimes granted custody (16). In the United States, fathers who murder the mother of their children face almost no barrier to obtaining custody (family courts are disconnected from criminal courts) after their prison sentences (17). As a result of these family court practices, violent perpetrators may seek custody of their children as a favorable way to regain respectability in society or to exculpate themselves of their crimes—often without having to change their behavior.

## The harm

Predictably, violent fathers who are granted custody often go on to abuse and murder their children at alarming rates. The United Kingdom has, through legislation, forced family courts to open to such research, but still found resistance. Deaths are the extreme end, but other harms also occur. For example, more than 58,000 children a year are ordered into their physical or sexual abuser's unsupervised custody following divorce in the United States (18). These children are likely to suffer “soul murder,” (19) or the psychological “death” that results from abuse. The consequences are lifelong psychological and physical problems and the loss of decades of life (20). The estimated economic loss in healthcare, child welfare, criminal justice, special education, and productivity, among other costs, has been estimated at $592 billion for 2018 (21). This is also an exponential problem, as almost 1 in 3 abused and neglected children become abusers themselves (22).

During the COVID-19 pandemic, the monitoring and reporting of child maltreatment were hampered further at a time of heightened need (23). In family courts, the “protective parent,” or the parent attempting to safeguard the child from abuse, often loses custody simply for alleging abuse. Sometimes, this occurs when the children allege the abuse. Separation from a protective, caregiving parent can have a similar effect as being orphaned; in the period when more than 140,000 children in the United States experienced the death of a parent or a grandparent caregiver from COVID-19 (24), close to 100,000 children were newly separated from a protective parent to be isolated with an abusive parent. Studies on family courts now show that, in addition to child deaths, protective parents, usually mothers, also disproportionately grow ill or die (25, 26).

## The pseudo-rationale

A source of these deaths is the regular practice of family courts of denying abuse, which is more likely to prolong cases for years and to require multiple court appointees whom the parents are obligated to pay (27). The revenue from doing so is estimated to be between $50 and $175 billion per year in the United States alone (28). In other words, there is a great financial incentive to place children in situations of greater danger and families in crisis.

The pseudo-rationale used to promote this result in the family courts is “parental alienation,” the notion that any allegation of abuse is a fabrication to “alienate” the other parent from the child and that any rejection on the part of the child is a result of “coaching.” However, research shows that while deliberate false reporting is rare [e.g., 0.1% in one study (29)], the abuse of children is not. In fact, child abuse is greatly underreported, a problem that has only been exacerbated during the pandemic (30). A national study of 4,388 custody cases showed that mothers who reported abuse—especially child sexual abuse—lost custody at alarming rates, sometimes to convicted sex offenders (31).

Multiple reputable scientific associations—including the United Nations (UN) (32)—have denounced family courts' use of the pseudo-concept of “parental alienation,” but it continues to be used almost ubiquitously. It is designed to punish and “turn the tables” against the victim to discourage the reporting of abuse. It enables courts to exculpate perpetrators of abuse so they not only escape prosecution, but are disproportionately rewarded with full custody, “child support,” legal fees, and even the incarceration of their victims in the form of a “debtors’ jail” if they cannot pay—operating without due process or the possibility of bail. In this manner, family courts amplify the “coercive control” patterns of domestic violence offenders, who typically establish their dominance through intimidation, isolation of their victims, and reversal of victim and offender (33). “parental alienation,” employed only within family court, not only contradicts established scientific, medical, and developmental research but enables severe and lasting trauma in children and adolescents through isolation and traumatic bonding with their abusers while separated from their primary supports (34).

## An international problem

A nationwide study found that family court custody decisions were overwhelmingly more wrong than right (35). Yet, an “abuse industry” has arisen around these wrong decisions, with its own group of lawyers, “officers of the court,” and poorly-trained “experts.” These arrangements are pecuniary: not only do abusers control the money the majority of the time, but they are also more willing to pay to avoid prosecution, while protective parents will more easily give up their savings to save their children's lives. Hence, in a manner even more pernicious than the “kids for cash” scandal (36), family courts are commodifying children and removing them from stable homes and protective, primary caregivers, at great harm, for greater profit.

This practice, unfortunately, has been exported worldwide. A journal’s special issue in 2020 first demonstrated the misuse of “parental alienation” in eight countries (37). Then, a number of international bodies began urging governments to consider testimonies of this approach as a “continuation of power and control” by abusive fathers (38). A European Parliament resolution soon “call[ed] on the Member States not to recognise parental alienation syndrome in their judicial practice and law.” (39) Moreover, a United Nations report found widespread family court practices of punishing mothers and children who brought forward credible allegations of abuse (40).

## Conclusion

Seldom brought under scrutiny, family courts have become a hidden, unchecked, domestic abusers’ tool for furthering their abuse. Children are the greatest casualties, who not only lose the opportunity to develop their potential, but for some, they become the next generation of substance users, rapists, and murderers, if they survive at all. Judicial abuses have increased and have received greater attention during the COVID-19 era, but attempts at reform, including legislative changes, have been marginally successful at best. Efforts to educate judges have been futile in the presence of enormous financial incentives to make decisions that are harmful for children. A culture of deadly abuse against the most vulnerable members of society calls for a moratorium on family courts in child custody decisions—along with the current structure of absolute judicial immunity, total secrecy, and lack of any accountability—to prevent the destruction of children by the very institutions intended to protect them. Child welfare matters should be handled outside of courts where possible and the rest should be dealt with in civil courts that have juries, due process, and transparency. Systemic changes are thus needed to preserve lives and better prevent exploitation of the most vulnerable members of society before the next pandemic.
